# Forskolin induces FXR expression and enhances maturation of iPSC-derived hepatocyte-like cells

**DOI:** 10.3389/fcell.2024.1383928

**Published:** 2024-04-17

**Authors:** Christiane Loerch, Leon-Phillip Szepanowski, Julian Reiss, James Adjaye, Nina Graffmann

**Affiliations:** ^1^ Institute for Stem Cell Research and Regenerative Medicine, Medical Faculty and University Hospital Düsseldorf, Heinrich Heine University, Düsseldorf, Germany; ^2^ IUF – Leibniz Research Institute for Environmental Medicine, Düsseldorf, Germany; ^3^ University College London, EGA Institute for Women`s Health- Zayed Center for Research Into Rare Diseases in Children (ZGR), London, United Kingdom

**Keywords:** induced pluripotent stem cells (iPSCs), hepatocyte-like cells (HLCs), Forskolin, cytochrome P450 activity, *in vitro* Differentiation

## Abstract

The generation of iPSC-derived hepatocyte-like cells (HLCs) is a powerful tool for studying liver diseases, their therapy as well as drug development. iPSC-derived disease models benefit from their diverse origin of patients, enabling the study of disease-associated mutations and, when considering more than one iPSC line to reflect a more diverse genetic background compared to immortalized cell lines. Unfortunately, the use of iPSC-derived HLCs is limited due to their lack of maturity and a rather fetal phenotype. Commercial kits and complicated 3D-protocols are cost- and time-intensive and hardly useable for smaller working groups. In this study, we optimized our previously published protocol by fine-tuning the initial cell number, exchanging antibiotics and basal medium composition and introducing the small molecule forskolin during the HLC maturation step. We thereby contribute to the liver research field by providing a simple, cost- and time-effective 2D differentiation protocol. We generate functional HLCs with significantly increased HLC hallmark gene (*ALB, HNF4*α*,* and *CYP3A4*) and protein (*ALB*) expression, as well as significantly elevated inducible CYP3A4 activity.

## Graphical Abstract

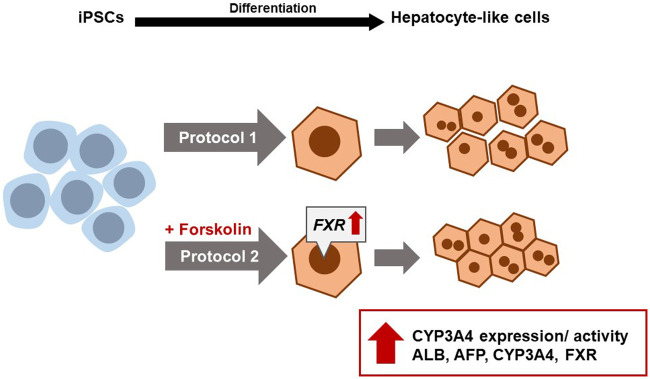

## 1 Introduction

Precise liver (-disease) models are indispensable considering society’s burden of emerging liver diseases (Asrani et al., 2019). However, to date, none of the available models is able to recapitulate human liver physiology accurately. Rodents show differences in the basal metabolic rate and cytochrome P450 activity compared to humans, implying an insufficient translation of rodent models to humans (Demetrius, 2004; 2005; Martignoni et al., 2006). Besides available tumor-derived cell lines such as HepaRG and HepG2, primary human hepatocytes (PHHs) are currently the gold standard for hepatocyte research. However, apart from their relatively high costs and ethical limitations, cultivation of the cells results in dedifferentiation within 24 h, thus attenuating their utility for disease modeling or analyses involving longer incubation (Godoy et al., 2016). In addition, donor-to-donor variation in their CYP genotype limits the reproducibility of results obtained with these cells.

Human induced pluripotent stem cells (iPSCs) and their differentiation potential to various cell types represent the future source for disease models. Patient-derived iPSC lines allow the consideration of specific genetic background. On the other hand, including several iPSC lines into a study, represents human genetic diversity more accurately and thus provides higher quality data compared to tumor-derived cell lines. Working with more than one iPSC line enables researchers to detect disease-associated factors, which span the genetic variance between patients.

Protocols differentiating iPSCs into hepatocyte-like cells (HLCs) were first established in 2007–2008 (Cai et al., 2007; Agarwal et al., 2008; Hay et al., 2008a; Hay et al., 2008b), and scientists have so far established various HLC differentiation protocols adapted to their needs. The 2D differentiation process takes about 3 weeks and provides a feasible tool to generate i) a HLC model for studying basic liver functions as well as ii) models to study the hepatocytes’ specific contribution to inherited and acquired diseases and iii) to test drugs. However, so far, the 2D HLC differentiation mostly results in rather immature HLCs resembling fetal hepatocytes (Baxter et al., 2015; Graffmann et al., 2022). As HLCs are mainly used for the study of diseases and drug development, HLC publications rather focus on a specific disease phenotype rather than the differentiation outcome itself. However, improving the differentiation protocols to consistently generate more mature iPSC-derived HLCs is indispensable for liver research.

In this study, we present our simple, cost- and time-efficient 2D differentiation protocol, optimizing our previously published protocols (Jozefczuk et al., 2011; Graffmann et al., 2016; Matz et al., 2017; Graffmann et al., 2021). By i) fine-tuning the cell number, ii) replacing penicillin/streptomycin with doxycycline, iii) using DMEM/F12 instead of L15 as a medium basis in the last step, as well as iv) adding the small molecule forskolin during the HLC maturation stage, we considerably increased stability of the process as well as maturation of the derived cells. This improved method provides a feasible protocol generating iPSC-derived HLCs within 18–20 days, showing a significant increase of HLC-hallmark gene and protein expression, enhanced functionality and improved uniformity of cell morphology. Since the generated HLCs show inducible activity of the adult enzyme CYP3A4, our model provides an interesting tool for drug development or disease modelling.

## 2 Methods

### 2.1 Ethical approval and human iPSC-Cultivation

The use of the human iPSC lines was approved by the Ethical Committee of the medical faculty of Heinrich Heine University, Düsseldorf, Germany, approval number: 5,704 and 5,013. Cell lines 2, 3 were kindly provided by Andrea Rossi, IUF–Leibnitz Institute für umweltmedizinische Forschung GmbH.

Urine-derived human iPSCs from a 51-year old healthy African male (Bohndorf et al., 2017), were cultivated on Matrigel (Corning) coated cell culture dishes with mTesR Plus (Stemcell Technologies) and penicillin/streptomycin (P/S) (Gibco). They were sub-cultivated in clusters by incubating with PBS w/o Ca^2+^, Mg^2+^ for 3–5 min at RT before mechanical detachment with a cell spatula and centrifugation at 40 *g* for 3 min. Before differentiation, the cells were sub-cultivated as single cells by incubating them with StemPro Accutase Cell Dissociation Reagent (Life Technologies) for 4 min at 37°C and subsequent centrifugation at 110 *g* for 4 min.

To show improvement of a protocol you need more than a single line-this is a major weakness!

### 2.2 HLC-differentiation

Human iPSCs were differentiated according to two distinct protocols, in the following referred to as protocol 1 (original) and protocol 2 (optimized), respectively. Protocol 1 is based on the protocol by Matz and Graffmann (Graffmann et al., 2016; Matz et al., 2017; Graffmann et al., 2021) with minor changes: Approximately 0.521 × 10^5^ iPSCs/cm^2^ were seeded onto Matrigel (Corning) coated plates. The medium was changed to Definitive Endoderm (DE) medium containing 96% RPMI supplemented with 2% B27 (w/o retinoic acid) 1% GlutaMAX (Glx) and P/S (all Gibco) and refreshed daily for the following 3 days. On the first day, 100 ng/mL Activin A (Peprotech) and 2.5 µM CHIR99021 (Stemgent) were added. For the next 2 days, the DE medium was supplemented only with 100 ng/mL Activin A. The medium was changed for hepatic endoderm (HE) induction to 77.5% DMEM/F12 supplemented with 20% Knockout Serum Replacement (KOSR), 0.5% Glx, 0.01% 2-Mercaptoethanol and 1% P/S (all Gibco). The medium was changed daily and 1% DMSO (Sigma-Aldrich) was freshly added for each medium change. After 4 days of HE medium, HLC medium was fed for 12–15 days, with medium changes every other day, consisting of 82% Leibovitz´s L-15 medium (Life Technologies), 8% FBS, 8% Tryptose Phosphate Broth (TPB), 1% Glx, 1% P/S (all Gibco). 1 μM insulin (Sigma-Aldrich), 10 ng/mL hepatocyte growth factor (HGF) (Peprotech), 25 ng/mL dexamethasone (Dex) (Sigma-Aldrich), and 20 ng/mL recombinant human Oncostatin M (rhOSM209a.a) (Immunotools) were freshly added to the medium.

In order to optimize the protocol, we adapted the original to generate protocol 2 as follows. We seeded 1.04 × 10^5^ iPSCs/cm^2^ onto Matrigel coated dishes. DE- and HE-induction were carried out as previously mentioned, but instead of P/S, 2 µM doxycycline was used. For the HLC medium we used DMEM/F12 as basal medium instead of Leibovitz´s L-15 (L-15) and 2 µM doxycycline instead of P/S. Insulin and growth factors remained, however, 20 µM forskolin (Tocris) was added freshly to the medium for every feeding.

### 2.3 Immunocytochemistry

Immunocytochemistry was performed on 4% PFA fixed cells after 10 min permeabilization with 0.5% Triton-X-100 (Sigma-Aldrich) in PBS. The cells were blocked with 3% BSA/PBS and subsequently incubated with primary antibodies against AFP, Albumin (both Sigma-Aldrich), HNF4α (Abcam) and E-CAD (Cell Signaling Technologies) overnight at 4 °C. Afterwards the cells were washed 3x with 0.5% Triton-X-100/PBS and incubated with fluorescence labeled secondary antibody against respective host species IgG (Life technologies) for 2 h at RT. DNA was stained with Hoechst 33258. Photomicrographs were taken with a LSM 700 microscope (Zeiss) and processed with ZEN software (Zeiss). Antibodies with their corresponding catalogue numbers and dilutions in Supplementary Table S1.

### 2.4 Western blot

Cells were lysed for 20 min on ice in RIPA-buffer containing protease- and phosphatase inhibitor (Sigma-Aldrich). Remaining cellular debris was centrifuged at 20,000 xg for 20 min at 4°C, and the supernatant was stored at −80 °C. Pierce BCA Protein Assay (Life Technologies) was performed to determine protein concentration as described by the manufacturer. 20 μg of protein was resolved on a NuPAGE™ 4%–12%, Bis-Tris protein gel (Life Technologies). Proteins were wet-blotted onto a 0.45 µm nitrocellulose membrane (GE healthcare, Solingen, Germany) and blocked in 5% Milk (ROTH) in TBS-T buffer. Afterwards primary antibodies against AFP and Albumin and Beta-actin (Cell Signaling Technologies) as housekeeping protein were incubated for 2 h at RT. After 3x washing with TBS-T, Horseradish peroxidase (HRP)-coupled secondary antibodies against respective host IgG (Cell Signaling Technologies) were incubated for 1 h at RT and non-bound antibody was washed off 3x with TBS-T. HRP chemiluminescence was detected using the 1x Pierce ECL Western blotting Substrate (Life Technologies) on a Fusion FX instrument (PeqLab). Corresponding band sizes were detected using the PageRuler pre-stained protein ladder (Life Technologies). Quantification was performed using Fusion Capt Advance software (PeqLab) and rolling ball background correction (Image Studio light 5.2).

### 2.5 Real-time quantitative PCR

RNA was isolated from HLCs, using the direct-zol RNA isolation kit (Zymo Research), following the manufacturer’s instructions. 500 ng of RNA were reversely transcribed to cDNA using the TaqMan reverse transcription kit (Life technologies). RT qPCR was performed in technical triplicates, using the Power Sybr Green Mastermix (Life technologies) and the VIIA7 machine (Life technologies). Primers were ordered from Eurofins. The mRNA expression was calculated as the log2-fold change relative to the housekeeping gene RPL0. Experiments were carried out in biological triplicates and two-tailed unpaired Student’s t*-*test was performed to calculate significances (please find primer sequences in the Supplementary Table S1). A 1% agarose gel (1x TBS buffer) was stained with GeldRed Nucleid Acid Gel Stain (Hoezel) and qPCR products were resolved at 100 V for 45 min.

### 2.6 Urea production

Urea Assay (QuantiChrom) was performed following the manufacturer`s instructions for low urea-samples. In brief, supernatant was collected from 3 independent differentiations and stored at −20°C. Urea standard was prepared in a 1:10 dilution, and 50 µL of samples were incubated with 200 µL of the reaction solution for 50 min at RT in the dark. Afterwards, emission was measured at a wavelength of 430 nm using the Epoch2 microplate reader (BioTek) and the concentration was calculated from the standard curve. Two-tailed unpaired Student’s t*-*test was performed to calculate significance.

### 2.7 Cytochrome P450 activity

CYP3A4-activity was measured using the P450-Glo™ kit (Promega), following the manufacturer’s instructions. In brief, cells were incubated with 1:1000 luciferin-IPA in Williams’ E medium (Sigma-Aldrich) for 1 h at 37°C. An equal volume of detection reagent was added and incubated for 20 min at RT in the dark, before measuring the luminescence using a luminometer (Lumat LB 9507, Berthold Technologies). Two-tailed unpaired Student’s t*-*test was performed to calculate significance.

### 2.8 Glycogen-storage

Glycogen storage was analysed using periodic acid-schiff (PAS)-reaction (Sigma-Aldrich) on PFA-fixated cells. The cells were incubated with periodic acid solution for 5 min at RT before washing 3x with dH_2_O. Afterwards the cells were incubated with Schiff`s reagent for 15 min at RT and washed 3x with tap water. Hematoxylin solution, Gill No 3 was incubated for 90 s to counterstain the nuclei. Glycogen storage was documented using brightfield microscopy with the IX50 Olympus Microscope (Olympus).

### 2.9 Indocyanine green (ICG) uptake

To test for ICG uptake and release capacity, HLCs were washed 1x with HBSS (Sigma-Aldrich) and incubated for 20 min at 37°C with 0.5 mg/mL ICG (Sigma) in HLC basal medium. Cells were washed 1x with HBSS and ICG uptake was documented via brightfield microscopy using the IX50 Olympus Microscope (Olympus). To release ICG, HLCs were incubated with HLC basal medium for 5–6 h at 37°C. After washing 1x with HBSS, ICG-release was documented with brightfield microscopy.

## 3 Results

### 3.1 Morphology and protein expression

**FIGURE 1:**
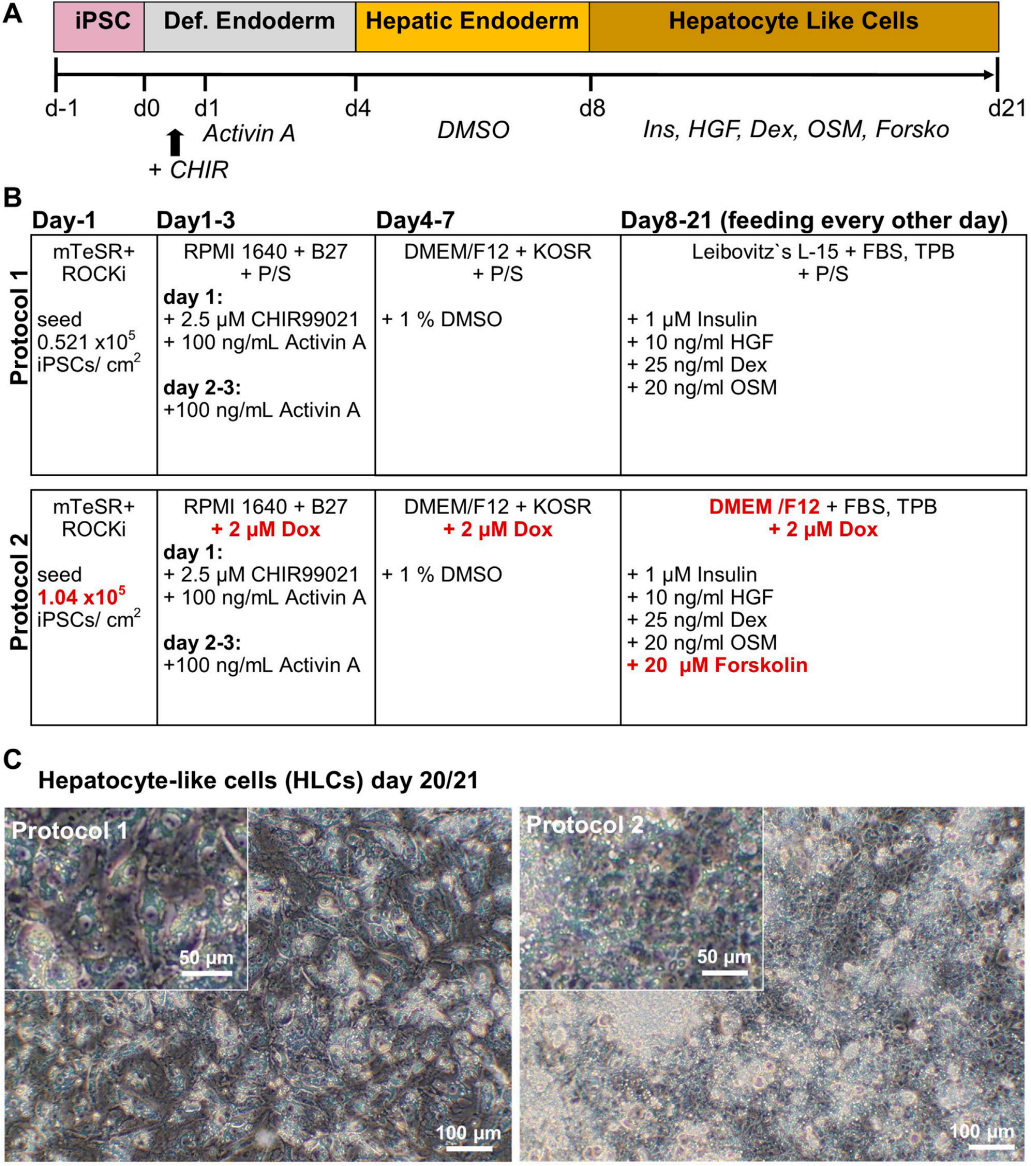
Generation of iPSC-derived hepatocyte-like cells (HLCs). **(A)** Time line for differentiation of iPSCs over 21 days, indicating Definitive Endoderm induction by CHIR99021 on d1, and Activin A treatment on d1-d3. Hepatic Endoderm was induced by adding DMSO on d4-d8. Maturation of HLCs was induced by insulin, hepatocyte growth factor (HGF), dexamethasone (Dex), oncostatin M (OSM) and forskolin (Forsko) treatment on d8-d21. **(B)** Comparison of protocol 1 and 2, changes in the media composition are marked in red. **(C)** Morphology of HLCs on day 20/21 generated with protocol 1 or 2.

**FIGURE 2:**
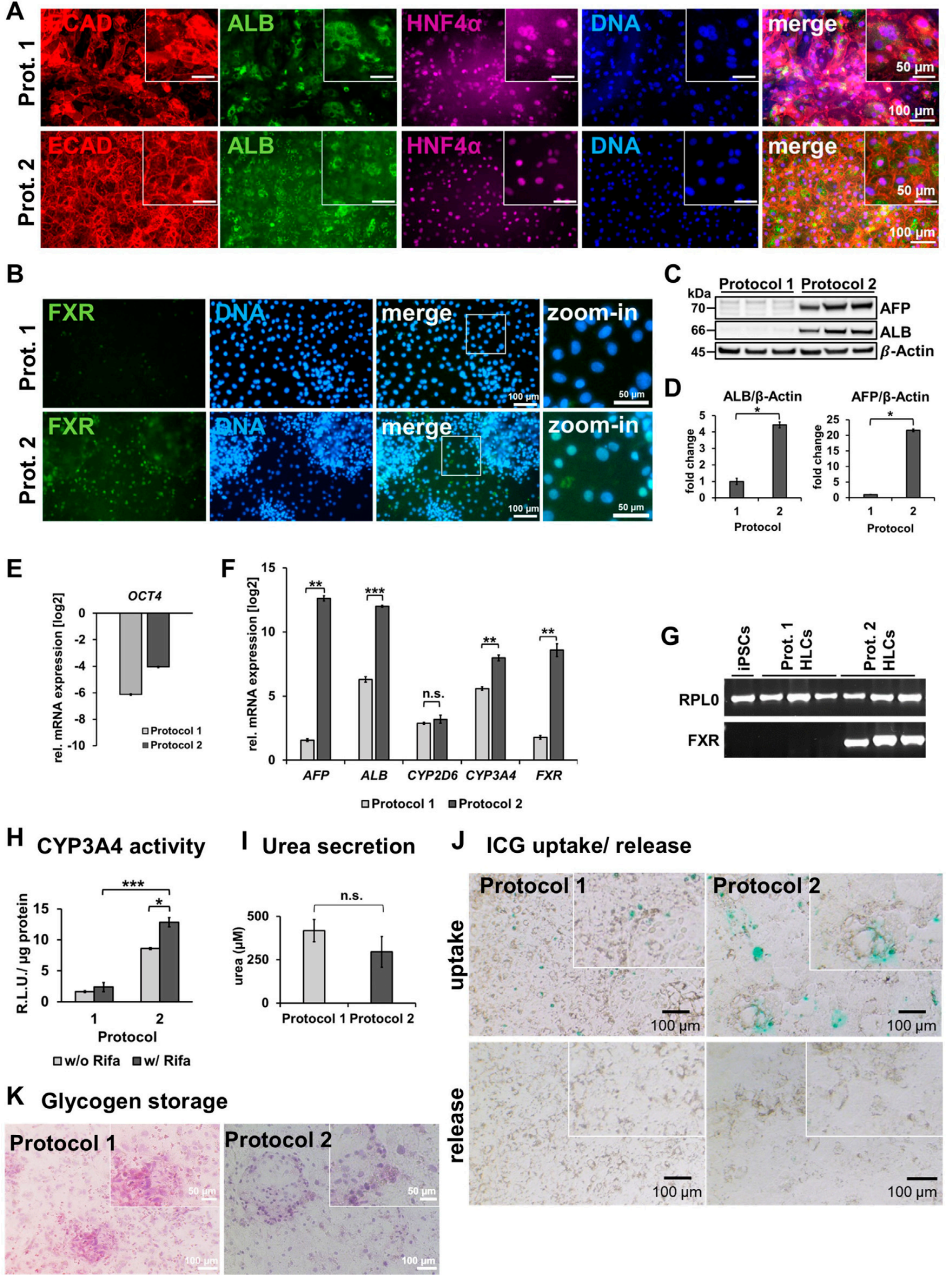
Characterization of HLCs generated with protocol 1 or protocol 2. **(A)** Representative immunofluorescence images of ECAD (red), ALB (green), HNF4α (purple) in HLCs differentiated with protocol 1 or two respectively. DNA was stained with Hoechst 33258. **(B)** Representative immunofluorescence images of FXR (green). DNA was stained with Hoechst 33258. **(C)** Western blots of AFP and ALB for 3 biological replicates of HLCs generated with protocol 1 or two respectively. **(D)** Fold change of protein expression (mean±SD) of three biological replicates of HLCs generated with protocol 1 or 2, normalized to housekeeping protein β-actin, relative to protocol 1. Two-tailed Student’s t-test was performed to calculate significances (*p < 0.05, **p < 0.01). Relative mRNA expression of **(E)** *OCT4* **(F)** *AFP*, *ALB*, *CYP2D6*, *CYP3A4* and *FXR* normalized to the housekeeping gene *RPL0* of HLCs generated with protocol 1 or two relative to iPSCs, respectively. Bar plots show mean± SE from three biological replicates. Two-tailed Student’s t-test was performed to calculate significances (*p < 0.05, **p < 0.01, ***p < 0.001). **(G)** Agarose gel electrophoresis showing bands of corresponding sizes indicating amplicons of housekeeping gene *RPL0* and *FXR* after RT-qPCR for iPSCs, and HLCs generated with protocol 1 or 2, respectively. **(H)** CYP3A4 activity determined by metabolization of luciferin-IPA as relative light units (R.L.U) normalized to total protein in HLCs generated by protocol 1 or 2 with and without rifampicin (Rifa) treatment for 24 h. Bar plots show mean±SD and significances were calculated by using two-tailed Student’s t-test (*p < 0.05, ***p < 0.001). **(I)** Secreted urea into the supernatant within 24 h. **(J)** Uptake and release after 6 h of indocyanine green (ICG) of HLCs generated with protocol 1 or 2. **(K)** Glycogen storage shown via Periodic Acid–Schiff (PAS) reaction on fixated HLCs generated with protocol 1 or 2 respectively.

iPSCs from a healthy 51-year-old African male were differentiated to hepatocyte-like cells (HLCs) using protocol 1 (original) or protocol 2 (optimized), resulting in cells showing different morphology and HLC properties (Figures 1A–C). Protocol 1 generated relatively large HLCs, while protocol 2 resulted in smaller, tightly packed HLCs showing more prominent borders (Figure 1C). Immunofluorescence staining shows the expression of the epithelial marker protein E-cadherin (E-CAD), and the hepatocyte markers hepatocyte nuclear factor 4alpha (HNF4α), and albumin (ALB) of resulting HLCs (Figure 2A). We detected all three markers in HLCs generated with both protocols, however a tendency towards a higher number of HNF4α-positive HLCs was visible in cells generated with protocol 2. ALB was expressed in HLCs generated with both media, and no significant difference could be detected in the immunofluorescence. However, Western blot analysis (Figure 2C) of HLCs and corresponding quantification (Figure 2D) detected significantly more AFP and ALB in HLCs generated with protocol 2 compared to protocol 1. Furthermore, we detected farnesoid X receptor (FXR) solely under optimized conditions. ICC confirmed its location to the nucleus of HLCs while in protocol 1 only unspecific background staining was detected (Figure 2B). FXR is a nuclear hormone receptor, involved in liver metabolism (Repa and Mangelsdorf, 2000; Sinal et al., 2000). Expression of FXR in iPSC-derived HLCs was recently found *in vitro* to be associated with increased maturity as observed in PHHs (Nell et al., 2022).

### 3.2 Gene expression and functionality

To confirm successful differentiation of iPSCs into HLCs, we measured relative mRNA expression of selected markers (Figure 2F). We observed a drastic decrease in *OCT4* levels compared to undifferentiated iPSCs (Figure 2E), indicating the loss of pluripotency in HLCs. Differentiation with protocol 2 resulted in a significant increase of gene expression of HLC markers *AFP*, *ALB*, and *CYP3A4* compared to protocol 1 (Figure 2F). Interestingly, we could show for *ALB* and *AFP* that this increase is directly linked to forskolin treatment, as HLCs differentiated according to protocol 2 but without forskolin, had a much lower expression of both factors (Supplementary Figure S1A). While AFP is considered an immature, fetal marker and ALB is also an early marker for HLCs, CYP3A4 is considered a late marker for adult hepatocytes, indicating mature HLCs (Martínez-Jiménez et al., 2007; Ang et al., 2018). We detected FXR expression only under optimized conditions (Figures 2F,G).

With regards to functionality, we could measure a significant increase of CYP3A4 activity in cells derived with protocol 2 compared to protocol 1 (Figure 2H). Importantly, CYP3A4 was inducible upon rifampicin treatment in protocol 2 cells, confirming their higher level of maturity. Again, this effect was linked to the forskolin treatment, as CYP3A4 as well as CYP2D6 activity was drastically reduced when culturing cells according to protocol 2 but without forskolin (Supplementary Figure S1B). We further investigated the effects of the optimized protocol with regards to urea secretion (Figure 2I), uptake and release of indocyanine green dye (ICG) (Figure 2J), and glycogen storage (Figure 2K). All HLCs secreted urea into the medium indicating key features of functional hepatocytes, however, we could not detect a significant increase with the optimized medium. ICG uptake was visible for both conditions, however only HLCs generated by the optimized medium were able to release ICG after 6 h of incubation. HLCs generated from both protocols stored glycogen. Furthermore, we noticed less cell death at the DE stage as well as through the transition from DE to HE stage. This might have also contributed to the enhanced maturation and homogeneity, which we observed at the HLC stage. However, as we did not test cell viability, our observation are based on a subjective impression.

## 4 Discussion

### 4.1 Fine-tuning of cell number and use of doxycycline improves HLC differentiation outcome

Differentiation of iPSCs to various cell types is only possible to a limited level since *in vitro* conditions cannot yet meet the delicately orchestrated balance of chemical and mechanical stimuli which are provided during the development *in vivo* (Ang et al., 2018). Differentiation of cells towards DE primes them for further HLC differentiation. In order to induce Activin/Nodal signaling, we use high concentrations of Activin A to activate the SMAD2/3 branch of the TGFβ pathway, which is essential for successful DE induction (Brown et al., 2011; Lee et al., 2011). Furthermore, WNT-signaling is needed to express SRY-box transcription factor 17 (SOX17) which upregulates several genes of the DE lineage. We use CHIR99021 to inhibit Glycogensynthase-Kinase 3 (GSK-3) and prevent β-catenin degradation, leading to the induction of SOX17 expression (Engert et al., 2013).

Especially during the first day of CHIR99021-and Activin A treatment, a lot of cell death has been observed, probably due to the drastic change in medium and Activin A and CHIR concentration (Peaslee et al., 2021). Nonetheless, after the first wave of cell death, massive cell proliferation takes place, demanding fine-tuning of the initial cell number. When the cell density is too low at the end of DE-stage, HE cells transit to an endothelial derived epithelial cell type, which is incapable of differentiating to HLCs (Graffmann et al., 2018). Therefore, we recommend testing the initial cell number for each cell line, as the DE outcome is indispensable for successful further differentiation. Peaslee et al. found that doxycycline can help to prevent apoptosis during the first Activin A incubation (Peaslee et al., 2021). Even though there was no direct effect on HLC-marker expression, they saw a general improvement in their HLC-culture with a more defined monolayer and better viability. This is consistent with our observation of lower cell death during DE-stage and enhanced HLC marker gene expression and functionality, when using doxycycline throughout the whole differentiation. However, we could not observe any direct effect on the DE-stage development.

During the HE-stage, numerous groups use 1% DMSO in a relatively plain basal medium to initiate hepatic endoderm development, though the direct mechanism of DMSO-induced differentiation is not yet completely resolved (Graffmann et al., 2022). In the past, we occasionally encountered a lot of cell death during the transition from DE to HE stage, leading to insufficient cell confluence and interference with the differentiation process. Although we did not analyze the direct impact of doxycycline on cell viability, we observed a lot less cell death during the transition from DE to HE. After 4 days of HE medium, we observed cobblestone shaped, tightly packed, polygonal HE-cells, expressing HNF4α and AFP.

### 4.2 Replacing L-15 medium with DMEM/F12 improves the maturation step

Many early hepatocyte differentiation protocols, including our protocol 1, used L-15 medium as a base for the maturation medium. It was introduced by Hay and others in 2008 (Hay et al., 2008a; Hay et al., 2008b) who based their maturation medium on a primary rat hepatocyte medium (Mitchell et al., 1984). L-15 medium is a phosphate and amino acid-buffered formulation, optimized for CO_2_-free culture conditions. In contrast, the media used in preceding steps of HLC differentiation (RPMI1640, DMEM), are sodium bicarbonate-buffered formulations, requiring 5%–10% CO_2_ for physiological pH of the culture medium. Cultivating cells in the L-15 based maturation medium in the same incubator utilized for the first steps of hepatocyte differentiation, results in a strong reduction of pH in the culture medium by exceeding the buffer capacity. This impairs cell survival, maturation, and function. Today, L-15 medium has been replaced by many groups with other media such as HepatoZYME from Gibco (Cameron et al., 2015) or hepatocyte basal medium from Lonza (Hannan et al., 2013). In our hands, DMEM/F12 (Sauer et al., 2016) turned out to be a reliable and cost-effective basal medium for successful HLC maturation.

### 4.3 Forskolin possibly induces HLC maturation via FXR and PXR pathway

The maturation phase of the differentiation is based on HLC promoting growth factors and the hormone insulin. Hepatocyte growth factor (HGF) is one of the essentials on the way towards HLC-differentiation since it is a morphogen and governs the development and regeneration of liver tissue via tyrosine kinase activity on its receptor C-MET (Bottaro et al., 1991; Graffmann et al., 2022). Oncostatin M, a cytokine which belongs to the interleukin six family and secreted by hematopoietic cells during embryonic liver development *in vivo*, is controversially discussed in the field. While some research groups found that it helps in the maturation of HLCs, it was also observed that it dedifferentiates HLCs, and is associated with cancers of several organs (Kamiya et al., 2001; Danoy et al., 2020; Graffmann et al., 2022). In our hands, the addition of OSM did not seem crucial for HLC development in the 2D culture, however, it mitigated the formation of cysts and prevented cell death in 3D (own unpublished data).

Using 1 µM of insulin for the HLC maturation has worked well for us. However, the concentrations vary throughout published protocols, and the exact function of insulin during hepatic differentiation is yet to be fully characterized. In fact, working groups have shown that manipulating energy metabolism of hepatic cells via insulin signaling might affect the differentiation outcome (Rodrigues et al., 2022).

Forskolin is a diterpene, found in the root of Coleus forskohlii (Dubey et al., 1981). The compound has been used in traditional medicine to treat hypertension, obesity, and respiratory diseases and its relevance for modern medicine is emerging (Lindner et al., 1978; Baumann et al., 1990; Jagtap et al., 2011; Sapio et al., 2017). On the biochemical level, it is known to upregulate adenylate cyclase which synthesizes cAMP from ATP (Seamon et al., 1981). cAMP promotes hepatocyte specific gene expression (Ogawa et al., 2013) and increases polarization of HepaRG cells via activation of farnesoid X receptor (FXR) and pregnane X receptor (PXR) (Mayati et al., 2018). This is in accordance with our detection of FXR-expression solely in the forskolin-containing protocol 2. Moreover, using protocol 2 without forskolin resulted in reduced levels of *ALB* and *AFP* expression as well as lower levels of CYP3A4 and CYP2D6 activity (Supplementary Figure S1A,B), demonstrating the important, yet elusive role of forskolin during HLC development.

FXR is a nuclear hormone receptor whose natural ligands are liver metabolites such as bile acids. While under physiological conditions it is involved in the clearance of bile acids and liver metabolism, its *in vitro* induction by using potent ligands leads to upregulation of HLC specific genes (Ali et al., 2015; Godoy et al., 2016; Stofan and Guo, 2020). Recently, Nell et al. demonstrated that FXR expression is essential for fostering hepatocyte fate while simultaneously inhibiting intestinal fate during HLC differentiation (Nell et al., 2022).

PXR is another nuclear hormone receptor, which is an environmental sensor of xenobiotics as well as endogenous ligands such as bile acids. Similar to FXR, it builds a heterodimer to activate transcription factor activity. As it is a chemical sensor for the liver environment it plays a pivotal role in diseases affecting the bile acid (BA) metabolism such as cholestatic disease, where it downregulates the BA synthesis and upregulates its export (Sayaf et al., 2021). Furthermore, recent studies also found an association with metabolic, inflammatory and lipid metabolism disorders, thus indicating a role for the metabolism of hepatocytes (Lehmann et al., 1998; Moreau et al., 2008; Godoy et al., 2016; Hakkola et al., 2016).

With FXR and PXR functioning as nuclear receptors with transcriptional activity on the metabolism of hepatocytes, it is plausible that activation of FXR and PXR via forskolin mediated cAMP-signaling promotes liver specification during differentiation *in vitro* (Godoy et al., 2016; Nell et al., 2022).

In conclusion, our data show a drastic increase in liver specific gene and protein expression such as AFP, HNF4α, ALB, CYP3A4 as well as the induction of FXR expression. Notably, protein and gene expression of AFP and ALB were highly increased in protocol 2 compared to protocol 1. However, as both are early markers of HLC development, they can only be considered as part of the HLC confirmation. Next to other functionality tests such as urea secretion, glycogen storage and ICG uptake and release, we could detect improved CYPP3A4 activity as well as rifampicin inducibility. Together with the increased levels of AFP and ALB, we could show a drastic optimization of our previous protocol. Furthermore, we could observe less cell death during the early steps of differentiation and enhanced viability and homogeneity of the monolayer at the HLC stage with the optimized differentiation protocol 2. However, more research is needed to further improve the efficiency of HLC differentiation. In particular, the fact that cells from both protocols still expressed residual *OCT4*, albeit with no significant differences between the protocols and on low levels, requires our attention, at least when aiming to generate HLCs for transplantation. Further analyses are necessary, to determine if this expression can be tracked down to single cells that could be removed by FACS sorting or if *OCT4* is still present in the majority of HLCs, which would require further optimization of the protocol. Nevertheless, especially the high and inducible level of CYP3A4 activity in cells derived with protocol 2 indicates an improvement in functionality, which is essential for using these cells for drug development and toxicological test.

Besides the addition of small molecules, the composition of the basal medium might also need further consideration. Replacing fetal bovine serum (FBS) with knock-out serum might be an aspect to consider, to move the protocol towards a xenofree setting which is essential for potential HLC transplantation and to reduce variability resulting from lot-to-lot variation. Similarly, Matrigel, a heterogenous matrix can be replaced with standardized and xenofree laminins as has been shown before (Cameron et al., 2015). Furthermore, using relatively high levels of insulin should be avoided as it manipulates the energy metabolism and might interfere with the differentiation efficiency (Hakkola et al., 2016). Differentiation in 3D enhances HLC culture stability compared to 2D which enables researchers to improve maturity and as well as to perform long-term experiments (Rashidi et al., 2018). This development suggests improvement for HLC differentiation in the future. To enhance reproducible maturity throughout the scientific community, it is indispensable to constantly update on the latest differentiation protocol. We therefore provide our current differentiation protocol, which robustly improved HLC hallmark gene expression and functionality.

## 5 Summary and outlook

The use of human iPSC-derived HLCs provides an urgently needed tool for future *in vitro* studies of liver diseases and therapy development. However, the established differentiation protocols are limited, because the resulting HLCs lack maturity (Graffmann et al., 2022). Companies offer differentiation kits which provide high levels of reproducibility and maturity, but are relatively expensive and thus not affordable for smaller laboratories. This underlines the need for protocols enhancing the maturation of HLCs available at reasonable costs. In this study, we introduce our optimized, simple and cost-efficient differentiation protocol, enhancing reproducibility and maturity of HLCs by adapting the initial cell number, addition of doxycycline to prevent cell death and the use of forskolin, an indirect cAMP agonist, in a DMEM/F12 based maturation medium.

## Figures and Tables

**Table:** 

**h**	hour
**ALB**	Albumin
**AFP**	Alpha-Fetoprotein
**DE**	Definitive endoderm
**Dex**	Dexamethasone
**ECAD**	E Cadherin
**FXR**	Farnesoid X receptor
**Fig**	Figure
**Forsko**	Forskolin
**GSK-3**	Glycogen synthase kinase 3
**HBSS**	Hanks buffered salt solution
**HE**	Hepatic endoderm
**HGF**	Hepatocyte growth factor
**HLCs**	Hepatocyte-like cells
**HNF4a**	Hepatocyte nuclear factor 4alpha
**IgG**	Immunoglobulin G
**ICG**	Indocyanine Green
**iPSCs**	induced pluripotent stem cells
**kDa**	kilo Dalton
**min**	minutes
**OCT4**	octamer-binding transcription factor 4
**OSM**	Oncostatin M
**PFA**	Paraformaldehyde
**P/S**	Penicillin/Streptomycin
**PAS**	reaction periodic acid-schiff reaction
**PBS −/−**	Phosphate buffered saline without calcium and magnesium
**PXR**	pregnane X receptor
**prot**	protocol
**R.L.U**	Relative light units
**RPL0**	Ribosomal Protein Lateral Stalk Subunit P0
**SOX**	SRY-related HMG-box genes
**TBS-T**	Tris-buffered saline with Tween20
**TPB**	Tryptose phosphate broth
**WNT**	Wingless-related integration site

## Data Availability

The original contributions presented in the study are included in the article/Supplementary Material, further inquiries can be directed to the corresponding author.
