# Supplementary material for: Forskolin induces FXR expression and enhances maturation of iPSC-derived hepatocyte-like cells
Source: Front Cell Dev Biol. 2024 Apr 17;12:1383928. doi: 10.3389/fcell.2024.1383928 (PMC11061433; doi:10.3389/fcell.2024.1383928)
Supplement: Supplementary file 1 [file DataSheet1.PDF]

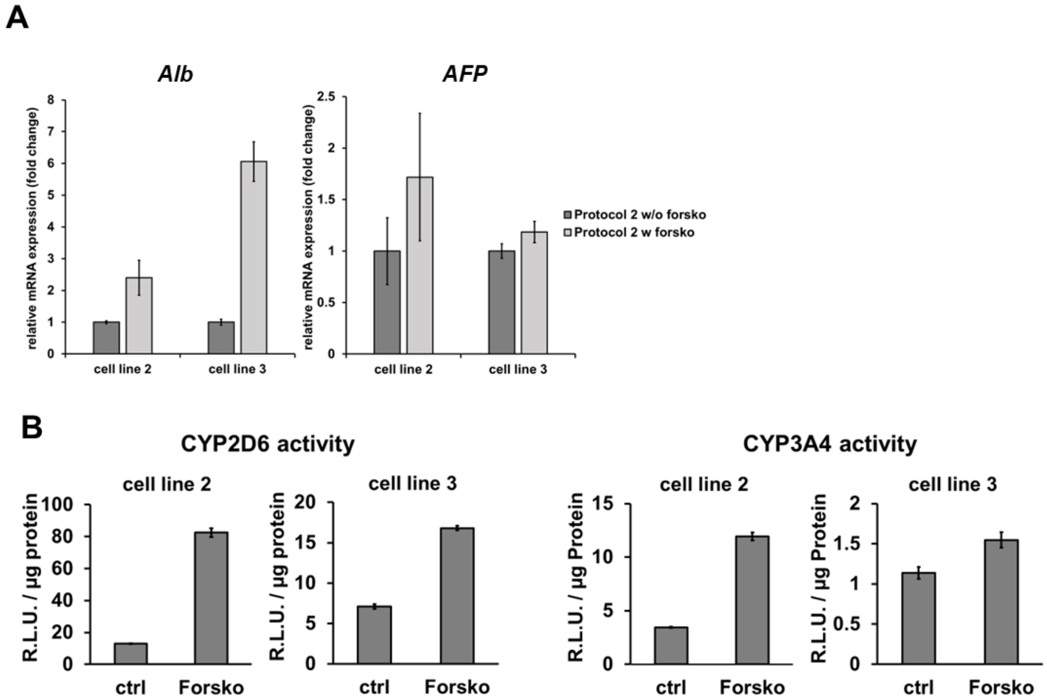

Supplementary Fig 1: Comparison of protocol 2 with or without forskolin. **(A)** Cell line 2 and 3 were differentiated according to protocol 2 with or without 20 µM forskolin (forsko). Relative mRNA expression of *Albumin* (*ALB*) and *Alpha-fetoprotein* (*AFP*) were measured in three biological replicates and normalized to the housekeeping gene *RPL0*. **(B)** CYP2D6 and CYP3A4 activity was measured using the respective kits from Promega in cell line 2 and cell line 3 derived HLCs when using protocol 2 with (Forsko) or without (ctrl) forskolin.
